# The American Medical Association—Section on Oral and Dental Surgery

**Published:** 1884-06

**Authors:** 


ARTICLE VII.

### THE AMERICAN MEDICAL ASSOCIATION —SECTION OF ORAL AND DENTAL SURGERY.

The meeting of this society at Washington has been largely attended this year, it being claimed that over thirteen hundred men from all parts of the country had registered as delegates.

The town seemed to be invaded by doctors, and the best hotels were packed until "standing room only" might have been displayed outside the dining rooms. Among so many men you must not be shocked if there were some who forgot their gentler duties amid the dizziness resulting from the novelties to them of the entertainments, scientific and gastronomic, the receptions, the excursions, and the refreshments. But few apartments were sufficiently large to comfortably contain the throngs from everywhere, and an irrepressible desire to get to the front was manifested by many who, no doubt, after experience and time have better schooled them, will be more humane and patient, if not more gentle and genteel. The courtesies and homage to the fair wives, daughters and ladies of the members' families were not overdone; in some cases, indeed, they were doubtless deferred to more convenient, if not more auspicious, opportunities. Alas, all men are human, and some at least who claim the M. D. are not lifted above their less skilled fellows in this world of many callings.

These strong contrasts, however, are the most notable exceptions, and if erudition, culture and perfection of manners do not always entwine in the one character, still, like the three graces, they rarely are pictured except together.

A few men of coarse minds and cruder cultivation will always float as a scum upon the top, which should be removed if a test of the general merit is to be applied.

The interest in the proceedings was very intense, and the results must necessarily be elevating to the profession as a whole. So important has the healing art become, that a division of the main body into sections seems to have been a necessity, and while this has uncovered many novelties of thought, the plan will yet require much executive ability to keep the powers within proper and profitable limits.

The running of a society of the size of this one, is by necessity only possible with machinery of such strength as to appear, when observed in relation to other societies, cumbersome and heavy. For the better management and smoother play of this machine, rings are largely introduced in its mechanism, and endless chains are not unfrequently formed by a junction of these. With proper application of oil and an occasional touch of plumbago, which is apt to be rubbed off by the unskillful engineers, the noise and friction are kept under subjection. Woe, however, to those who fail to attend these complicated duties. If the journals are heated, they at once show defects in the apparatus, and these are numerous and dangerous; for, the corrections of the faults are apt to involve a change of the engineers, whose only pay is the quenching of their ambition for offices, and the transient publicity that they derive from these positions of honor.

An example of the curious ways in which the general nominating committee performed its duties, was afforded by the Section of Oral and Dental Surgery having a chairman assigned it, who disclaimed all knowledge of the nomination, and upon the section proposing to take an informal ballot, he made some remarks that we construed to be equivalent to a resignation. For secretary there was given to the same Section one who it is claimed never had been to one of its meetings, and was not even present at this session. "*Sic transit gloria.*" The general association at its final Session passed a resolution, to be adopted as an amendment next year, that each section hereafter propose the names of its own officers to the committee on nominations.

Other sections were said to be under the management of parties adverse to the prominent members of the societies of specialists, of which there are several besides dentistry. In the latter it was believed by some that hope of election to high places in the American Dental Association was the only reason for the absence of those who would have gladly joined hands in the work there, if not intimidated by the possibility of its depressing the ardor of friends for their nomination in the A. D. A.

What pitiable poverty this petty planning proves, and how puny must be the outlook of the world to those who so preserve their perilous poise while posing as perfect patterns before their fellows. It is a shallow plasure indeed if such trifles can destroy it. Can it be that the time for the office to seek the man has altogether passed, even among professional men ?

The struggle for any advancement, save through honorably acquired ability, is so generally followed by a corresponding downfall, that one cannot but wonder at the blindness of men who would aspire to positions above their abilities to fill. If these defects are so apparent on the level of the floor, how much more conspicious would they be in the chair of the presiding officer.

There are likewise other serious troubles to be met in the very limited membership which the above mentioned section will admit of, there being but a dozen or so in attendance. Upon the part of these representatives there was a laudable manifestation of a determination "to raise up those who fall, and finally to beat down Satan under our feet." Papers were read that took high grade views of the duties of the dental surgeon as a member of the broad fraternity of the healing art, and the discussions were not lacking in boldness of conception of the proper methods of discharging the functions of this branch. At least this association may become another of the many means of advancement, and its infancy as yet, should shield it from too harsh criticism or hasty demands for lusty strength.

Hints were freely made as to the doubtful manner in which one of the delegates had obtained the right to be present, and if these were correct it cannot be too soon to expose an error that is by many conceded to be the bane of the dental profession of to-day. Degrees purchased by subscriptions of money to Colleges, or services rendered institutions weak in their powers of attraction to their fonts, will not alone render their possessors open to criticism, but likewise retard, if they do not degrade the Section's work by the reflection they may arouse as to the shallowness of the channels through which the right of membership may be attained. Perhaps an investigation might at least raise the confidence of some, as to the altitude of the standard reguired for the privileges of fellowship. So it will not be amiss to hope that these hints may be formulated into charges that will be brought through the proper channels to this association as a committee of the whole.

Agitation may endanger the life of the section, but what is the serving of that compared with the dues to truth and justice? Young trees are mostly benefitted by fearless and thorough pruning. Excresences of an unsightly nature had better be cut away before they disfigure the whole growth, and the wisest of men has given us the rule that to "spare the rod is to spoil the child."—T. C. S. in *Dental Practitioner.*

